# Successful Extension of Vascularized Composite Allograft Perfusion Cold Storage to 24 h in a Rat Hindlimb Transplant Model

**DOI:** 10.1097/TXD.0000000000001623

**Published:** 2024-05-15

**Authors:** Po’okela K. Ng, Dor Yoeli, Joy L. Huang, Yuhuan Luo, Yong Wang, Bing Li, Zhaohui Wang, Jesse Schold, Swati Jain, An-Jey A. Su, David W. Mathes, Kia M. Washington, Evan Farkash, Alkesh H. Jani, Christene A. Huang

**Affiliations:** 1 Department of Surgery, University of Colorado Anschutz Medical Campus, Aurora, CO.; 2 Department of Medicine, University of Colorado Anschutz Medical Campus , Aurora, CO.; 3 Department of Pathology, University of Michigan School of Medicine, Ann Arbor, MI.

## Abstract

**Background.:**

Vascularized composite allograft transplantation is a treatment option for complex tissue injuries; however, ischemia reperfusion injury and high acute rejection rates remain a challenge. Hypothermic machine perfusion using acellular storage perfusate is a potential solution. This study evaluated the University of Wisconsin Kidney Preservation Solution-1 (KPS-1) compared with normal saline (NS) for preservation of donor rat hindlimbs subjected to 24 h of ex vivo perfusion cold storage.

**Methods.:**

Hindlimbs were subjected to 24-h perfusion cold storage with heparinized KPS-1 (n = 6) or heparinized NS (n = 6). Flow, resistance, and pH were measured continuously. At the end of the 24-h period, tissue was collected for histological analysis of edema and apoptosis.

**Results.:**

KPS-1 perfused limbs showed significantly less edema than the NS group, as evidenced by lower limb weight gain (*P* < 0.001) and less interfascicular space (*P* < 0.001). KPS-perfused muscle had significantly less cell death than NS-perfused muscle based on terminal deoxynucleotidyl transferase dUTP nick-end labeling (*P* < 0.001) and cleaved caspase-3 staining (*P* = 0.045). During hypothermic machine perfusion, a significant decrease in pH over time was detected in both groups, with a significantly greater decline in pH in the KPS-1 group than in the NS group. There were no significant differences overall and over time in flow rate or vascular resistance between the KPS and NS groups.

**Conclusions.:**

Perfusion with KPS-1 can successfully extend vascularized composite allograft perfusion cold storage for 24 h in a rat hindlimb model without significant edema or cell death.

## Graphical abstract

**Figure:**
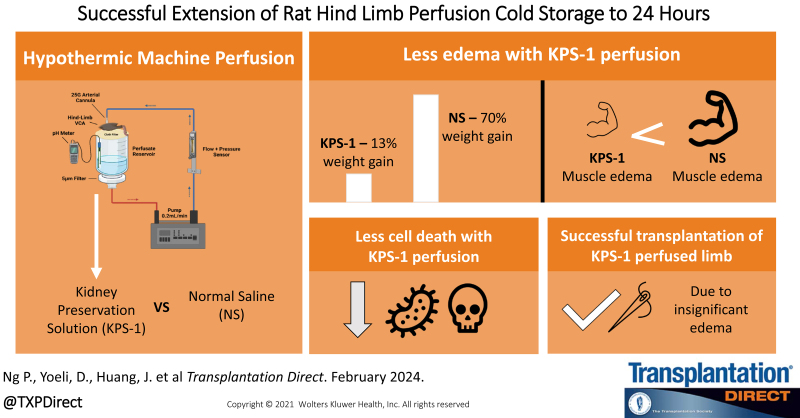

A vascularized composite allograft (VCA) consists of multiple tissues such as muscle, bone, nerves, and skin, intended for transplantation as a functional unit (eg, hand or face).^1^ VCAs serve as potential replacements for traumatic tissue loss, including limb injuries and limb losses from explosive devices in the battlefield.^2-6^ Clinical applications of VCA transplantation allow for the reconstruction and replacement of damaged or lost tissue with the same tissue from a suitable donor.^7^ VCA transplantation is not considered a lifesaving procedure; however, it serves to improve patient quality of life and is thus labeled a life-enhancing operation.^8^ This treatment is currently limited due to the high susceptibility of soft tissue to ischemia reperfusion injury (IRI) and lifelong immunosuppression requirement for patients, making recipients prone to opportunistic infections, chronic kidney failure, and many other long-term complications.^9-14^ To date, relatively few VCA transplants have been performed: approximately 150–200 VCA transplants worldwide.^15,16^ Currently, the acceptable ex vivo storage time is limited to 6 h, but the detrimental effects of prolonged ischemic storage can be observed as early as 3 h.^17-20^ As a result, some have advocated the transport of the donor and recipient to the same site to minimize cold ischemia time, which is often logistically challenging, if not impossible, with a multiorgan donor. Therefore, optimal cold storage strategies are required to improve VCA transplant outcomes and expand the geographical donor pool.

Hypothermic preservation solutions for VCAs have largely been adopted from previous experience in solid organ preservation for transplantation, such as in the kidneys and liver. The University of Wisconsin (UW) perfusion solution has been described as the preservation solution of choice during static cold storage of clinical VCA transplants.^21,22^ However, the effects of different preservation solutions have not been studied in clinical VCA transplantation, and there have been cases where heparinized saline solution has been used for VCA flushing and preservation.^22^

In addition to the solution itself, the best method of administration has been debated. While solid organ allografts can be submerged in preservation solution, there is concern for edema and skin maceration when submerging soft tissues such as VCAs. Thus, the current gold standard method of VCA preservation includes wrapping the tissue in gauze dampened with a preservation solution, which helps retain moisture in the tissue. Translational research has identified several potential therapeutic agents that can be delivered during cold storage to mitigate organ damage during the cold ischemia period and after reperfusion.^23,24^ However, the current method of static cold storage for VCAs significantly limits the amount of preservation solution that can be delivered to the allograft. Therefore, novel strategies to better deliver these therapeutic agents during cold storage are needed to translate these findings to the field of VCA transplantation.

Hypothermic machine perfusion (HMP) may be a strategy that allows for consistent and regulated circulation of preservation solution throughout VCA tissues by providing constant exposure without tissue submergence. It is well-documented that HMP storage can decrease IRI in kidney grafts, and it is considered the clinical gold standard for preservation of kidneys with expected prolonged cold storage time or grafts procured from marginal or extended criteria donors.^25-27^ A similar protocol has not yet been approved for clinical use in VCA transplantation. Research on VCA perfusion is limited and has largely focused on normothermic perfusion utilizing oxygenated blood as the perfusate.^28-30^ The need for an oxygen source and donor blood for perfusion increases the complexity, size, and cost of the perfusion machine, thereby limiting its clinical applicability, particularly in remote and/or small hospitals.

UW Kidney Preservation Solution-1 (KPS-1) is a modified form of UW solution with lower viscosity, making it ideal for use in allograft perfusion. It is the most common preservation solution used for kidney HMP and has the potential to be a preservation solution for VCA HMP. Studies investigating other perfusion solutions such as BSA- or DMEM-based perfusates have shown much shorter storage times and high rates of apoptosis, necrosis, and excessive edema; therefore, we chose an alternate perfusion solution that has already been established in the clinical setting for extending cold storage times in the setting of kidney transplantation.^11^ This study evaluated the ability of heparinized UW KPS-1 solution to preserve donor rat hindlimbs subjected to 24-h ex vivo cold perfusion storage without significant edema, compared with heparinized normal saline (NS).

## MATERIALS AND METHODS

### Study Cohort

Male Brown Norway rats purchased from Charles River Laboratories were used as the hindlimb donors. Animals were housed in groups of 2–3 per cage with free access to food and water. Bilateral hindlimbs were procured from 6 donor animals aged 14–18 wk. Limbs were subjected to 24 h of HMP using either heparinized KPS-1 or heparinized NS at 4 °C in a temperature-controlled room. All animal experiments were performed in accordance with the Institutional Animal Care and Use Committee of the University of Colorado Anschutz Medical Campus (Protocol Number 00851) and the US Army Medical Research and Development Command Animal Care and Use Review Office (Protocol Number RT190072P1.e001). All procedures followed the ethical guidelines for animal research.

### Hindlimb Procurement

Rats were anesthetized with an intraperitoneal weight-based injection of ketamine/xylazine. Once sedated, bilateral hindlimbs were shaved and prepped with betadine and alcohol. The femoral artery and vein were identified and isolated. The artery was dissected superior to the common iliac artery. Both vessels were then ligated and cut. The remaining muscle, skin, and bone were removed from the limb. The artery of the limb to be perfused was cannulated using a 25-gauge blunt needle cannula and secured using 9-0 nylon sutures. The artery was then flushed with 5 mL KPS-1 solution or NS solution containing 100 units/mL heparin. Each limb was kept on ice until transported to a 4 °C temperature controlled room. This procedure was repeated on the contralateral side. Following completion of the surgery, the donor was euthanized under anesthesia.

### Machine Perfusion Apparatus

**FIGURE 1.**
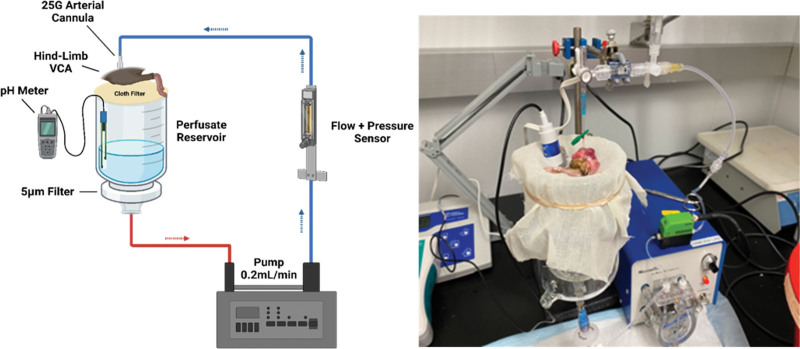
Representative diagram and image of the machine perfusion apparatus designed and used for 24-h hindlimb perfusion. Blue lines indicate flow into the limb, red indicates perfusate flow out of the limb. VCA, vascularized composite allograft.

Perfusion was achieved using a flow rate-controlled peristaltic pump (Figure 1). A reservoir of 50 mL of KPS-1 or NS each containing 4000 units of heparin was used as the perfusion solution. Effluent perfusate drained passively out of the femoral vein and into a reservoir, which fed the perfusate back into the circuit. A cloth filter was used to prevent macroscopic debris from entering the reservoir, and a 5-micron filter was placed between the reservoir and the pump to collect microscopic debris. Flow rate, pressure, and perfusate pH were continuously monitored. The pump was set to an initial target flow rate of 0.2 mL/min at the beginning of perfusion and was kept constant to observe changes in vascular resistance over time. Flow, resistance, and pH were measured hourly for 24 h, with hours 0–2 reserved for pH equilibration.

### Immunohistochemistry

After 24 h of cold storage, tissue samples were collected and submerged in 10% formalin for 24 h, then transferred and stored in 70% ethanol before paraffin embedding. Paraffin-embedded tissues were cut into 4 µm sections and slides were stained with hematoxylin & eosin (H&E), Terminal deoxynucleotidyl transferase dUTP nick-end labeling (TUNEL), and cleaved caspase-3 (CC3) (Cell Signaling No. 9661, Danvers, MA). Staining was performed for both treatment groups as well as nonperfused naive muscle tissue as a control according to standard/manufacture protocols (Nos. RMR622, RBR962; Biocare, Concord, CA).

### Histological Analysis

TUNEL and CC3 staining was quantified by counting the total number of positively and negatively stained cells in 5–10 random ×10 longitudinal fields to calculate the percentage of positively stained cells (Aperio ImageScope software; Leica Biosystems, Buffalo Grove, IL). H&E-stained slides were used to quantify edema in 5 random ×10 and ×20 longitudinal fields by measuring the intercellular and interfascicular space using linear intercellular distance and area (white space) in each limb, respectively (ImageJ software; National Institute of Health, Bethesda, MD).

### Statistical Analysis

Statistical analysis was performed using GraphPad Prism (Version 6.0; GraphPad Software, San Diego, CA) and SAS 9.4 (SAS Institute, Cary, NC). The data are expressed as mean ± SD unless otherwise stated. The pre-post change in weight within a group was tested using a paired *t* test, and the comparison between groups in the pre-post change was performed using a 2-sample unpaired *t* test. For HMP, we examined the association between both treatment groups (KPS-1 versus NS), time (h), and the interaction between treatment group and time with 3 response variables: flow (mL/min), vascular resistance (mm Hg/min/mL), and pH level. To account for repeated methods within subjects, we used linear mixed models to assess differences in overall levels, changes over time, and changes over time between the treatment groups. For these models, we considered individual subjects as random effects and treatment group, time, and treatment group by time as fixed effects. We iteratively tested the covariance structure of the random effect considering compound symmetry, unstructured, autoregressive, Toeplitz, and heterogeneous autoregressive and selected the models that minimized the Akaike information criteria. Statistical significance was set at *P* < 0.05.

## RESULTS

### Perfusion Apparatus

**FIGURE 2.**
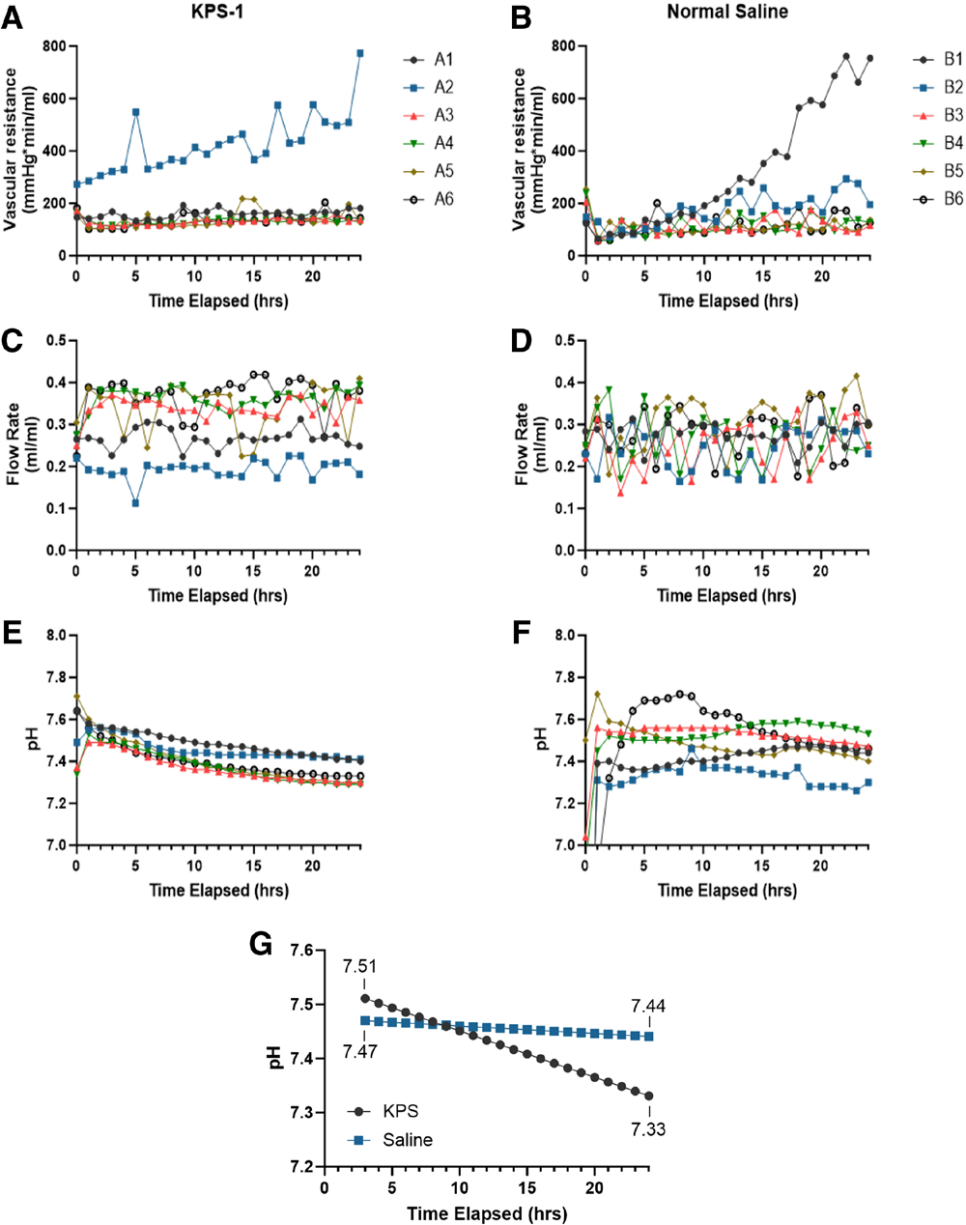
Perfusion parameters. Graphs showing hourly measurements of vascular resistance (A, B), flow rate (C, D), and pH (E, F) for each individual limb perfused with KPS (A, C, E: limbs A1–A6) or normal saline (B, D, G: limbs B1–B6). G, Results of the mixed model analysis comparing rates of pH change between each group. KPS-1, Kidney Perfusion Solution-1.

During HMP, hourly measurements of flow, vascular resistance, and pH were taken to analyze potential changes and ensure that constant perfusion was achieved throughout the entire 24-h period (Figure 2A–F). For both treatment groups, the mean and median flow level were 291.5 ± 68 and 293.9 μL/min (Q1 = 239.7, Q3 = 351.8), respectively. The mean and median vascular resistance were 173.3 ± 123.6 and 134.2 mm Hg/min/mL (Q1 = 109.2, Q3 = 168.5). The mean and median pH levels after 2 h were 7.44 ± 0.09 and 7.44 (Q1 = 7.36, Q3 = 7.50).

No statistically significant differences were detected between the treatment groups, time, or treatment groups by time with respect to both flow (Table 1) and vascular resistance (Table 2). For pH, significant associations were detected between treatment groups by time (Table 3); there was a significant decrease in pH during HMP with KPS-1 relative to NS (*P* = 0.008) (Table 3; Figure 2G). A 2-h equilibration period was used since the starting pH of NS is lower than KPS as shown in Figure 2F. The least squares mean estimates at hour 3 were 7.51 (SE = 0.03) and 7.47 (SE = 0.03) in the KPS and NS groups. At hour 12, the mean estimates were 7.43 (SE = 0.03) and 7.46 (SE = 0.03) in the KPS and NS groups. At 24 h, the mean estimates were 7.33 (SE = 0.03) and 7.44 (SE = 0.03) for the KPS and NS groups.

**TABLE 1.** Vascular resistance: results of mixed model for association of treatment group over time

Parameter	Level	Estimate	SE	*df*	*P * ^*a*^
Intercept		187.66	68.9	10	0.02
Treatment group	KPS	4.42	97.5	10	0.96
	Saline (reference)	–	–	–	–
Time	Hours	4.50	3.27	286	0.12
Treatment group^*a*^ time	KPS^*a*^ hour	–1.74	4.62	286	0.70
	Saline^*a*^ hour (reference)	–	–	–	–

^*a*^Result of type 3 F test of fixed effect: neither study group, time or the rate of change by study group were statistically significantly different.

KPS, Kidney Perfusion Solution.

**TABLE 2.** Flow rate: results of mixed model for association of treatment group over time

Parameter	Level	Estimate	SE	*df*	*P* ^*a*^
Intercept		265.43	20.76	10	<0.001
Treatment group	KPS	37.43	29.36	10	0.23
	Saline (reference)	–	–	–	–
Time	Hours	0.76	0.67	286	0.24
Treatment group^*a*^ time	KPS^*a*^ hour	–0.38	0.95	286	0.69
	Saline^*a*^ hour (reference)	–	–	–	–

^*a*^Result of type 3 F test of fixed effect: neither study group, time or the rate of change by study group were statistically significantly different.

KPS, Kidney Perfusion Solution.

**TABLE 3.** pH: results of mixed model for association of treatment group over time

Parameter	Level	Estimate	*df*	*P * ^*a*^
Intercept		7.47	0.03	<0.001
Treatment group	KPS	0.06	10	0.23
	Saline (reference)	–	–	–
Time	Hours	–0.001	0.001	0.35
Treatment group^*a*^ time	KPS^*a*^ hour	–0.007	0.002	0.008
	Saline^*a*^ hour (reference)	–	–	–

^*a*^Result of type 3 F test of fixed effect: no statistically significant difference in overall levels of pH were observed between study group or changes over time. However, a more rapid decline in pH was observed among the KPS group relative to the saline group.

KPS, Kidney Perfusion Solution.

### Edema

**FIGURE 3.**
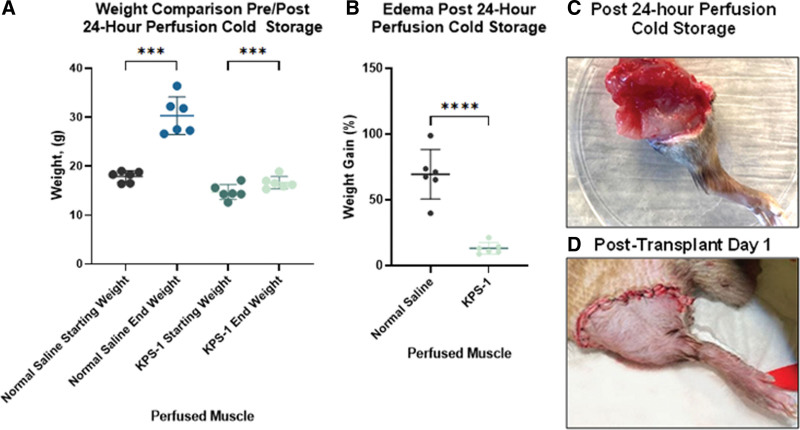
Limb edema (weight gain). A, Comparison of weight (in grams) before and after 24-h perfusion with normal saline or KPS. The pre-post change in weight within a group was tested using a paired *t* test (normal saline *P* = 0.0003; KPS *P* = 0.0001). B, Quantification of weight gained displayed as a percentage of weight gained relative to starting weight of hindlimbs (n = 12). The comparison between groups in the pre-post change was performed using a 2-sample unpaired *t* test (*P* < 0.0001). C and D, Representative images of Brown Norway hindlimbs that were subjected to 24 h perfusion with KPS solution ex vivo (top), and in vivo (bottom) following successful orthotopic transplantation. Significant differences, *P < 0.05, **P < 0.01, ***P < 0.0001. KPS-1, Kidney Perfusion Solution-1.

Limbs were weighed before and after 24 h of perfusion cold storage to approximate the extent of edema. The mean start- and end-weights of all limbs before and post-perfusion were 16.3 ± 2.0 and 23.5 ± 7.3 g, respectively. Therefore, a significant increase in limb weight gain was observed in both NS and KPS-1 perfused muscle at the end of 24 h (*P* < 0.001 for both) (Figure 3A). However, the average percentage of weight gain was significantly lower in KPS-1 perfused hindlimbs than in those perfused with NS (69.5% ± 0.17% versus 13.3% ± 0.04%; *P* < 0.001) (Figure 3B). Orthotopic hindlimb transplantation could successfully be performed with limbs perfused with KPS-1 (Figure 3C and D), but not with NS-perfused limbs due to excessive edema.

**FIGURE 4.**
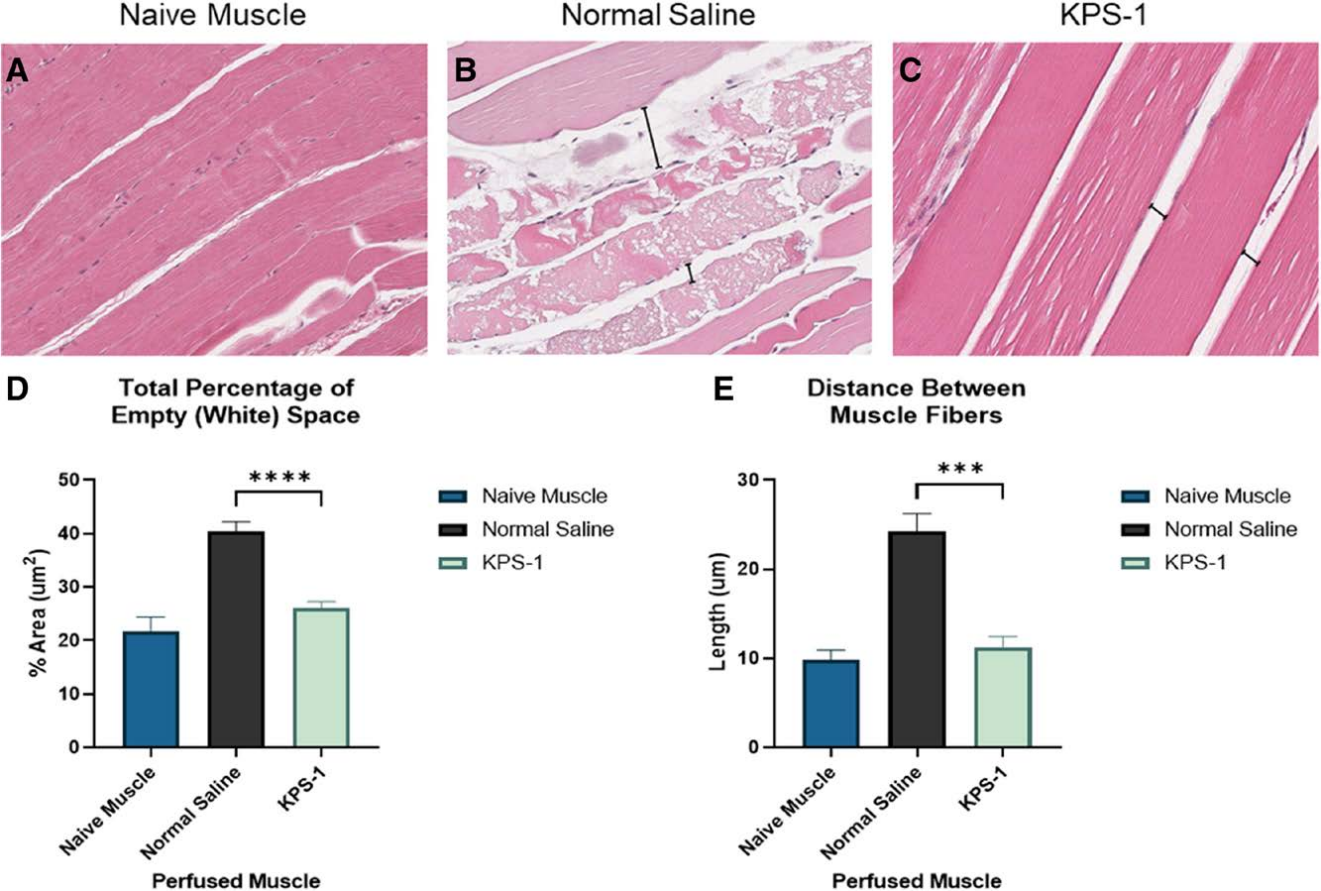
Histological assessment of edema. A–C, Representative ×10 H&E staining of Brown Norway rat hindlimb muscle. H&E of hindlimb muscle (left to right) naive untreated hindlimb, hindlimb perfused for 24 h with normal saline, hindlimb perfused for 24 h with KPS solution. D and E, Quantification of edema as a function of interfascicular space, (D) percent of empty space (*P* < 0.0001), and (E) length of gaps between skeletal muscle fibers (*P* = 0.0002) (see marker). The comparison between KPS and normal saline groups was performed using an unpaired *t* test. Significant differences, *P < 0.05, **P < 0.01, ***P < 0.0001. H&E, hematoxylin & eosin; KPS-1, Kidney Perfusion Solution-1.

To further study the effects of our perfusion cold storage system on hindlimb edema, H&E staining was performed. Skeletal muscle samples from both treatment groups and a control group consisting of nonperfused naive muscle tissue were stained to quantify the interfascicular space (Figure 4A–C). After 24 h of perfusion cold storage, hindlimb muscles perfused with KPS-1 (Figure 4D and E) showed significantly less edema than samples perfused with NS in both area (26.1 ± 2.9 versus 40.4 ± 4.6µm^2^; *P* < 0.001) and linear approaches (11.3 ± 2.9 versus 24.2 ± 4.9 µm; *P* < 0.001).

Furthermore, KPS-1 perfused samples did not demonstrate significant edema when compared with naive hindlimb muscle, but NS-perfused samples showed significant edema in comparison with naive samples (area: 40.4 ± 4.6 versus 21.8 ± 6.8 µm^2^; *P* < 0.001 and linear: 24.2 ± 4.9 versus 9.8 ± 3.02 µm; *P* < 0.001) (Figure 4D and E).

### TUNEL and CC3 Staining

**FIGURE 5.**
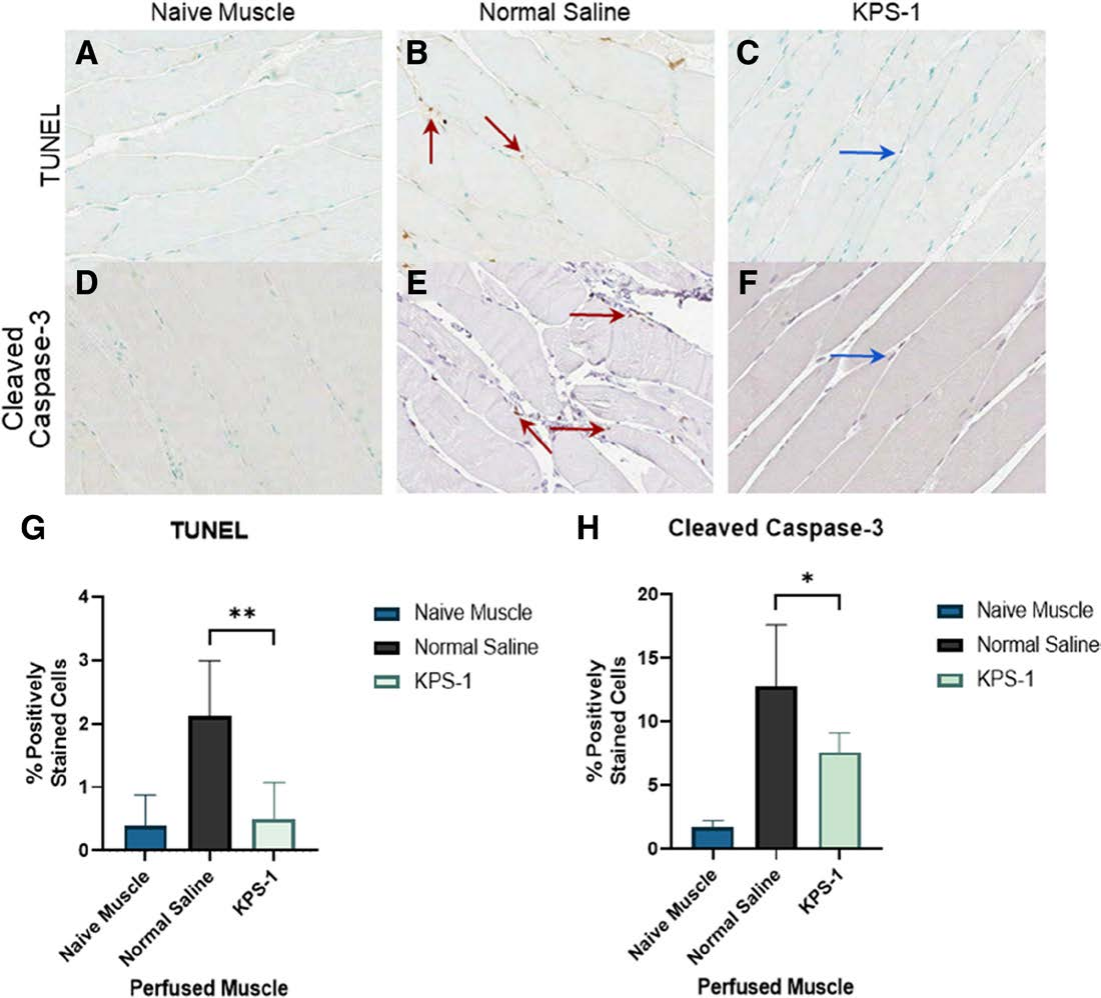
Representative ×10 TUNEL and ×10 cleaved caspase-3 staining images of rat hindlimb muscle, positive/negative staining indicated by red/blue arrows, respectively. TUNEL staining of hindlimbs: (A) naive muscle, (B) muscle perfused with normal saline, and (C) muscle perfused with KPS solution. Cleaved caspase-3 staining of hindlimbs: (D) naive muscle, (E) muscle perfused with normal saline, and (F) muscle perfused with KPS solution. Quantification of TUNEL (G) and caspase staining (H). The comparison between KPS and normal saline groups was performed using an unpaired *t* test. Significant differences, *P < 0.05, **P < 0.01, ***P < 0.0001. (TUNEL *P* = 0.0064; cleaved caspase-3 *P* = 0.044). KPS-1, Kidney Perfusion Solution-1; TUNEL, terminal deoxynucleotidyl transferase dUTP nick-end labeling.

The results of TUNEL staining (Figure 5 A-C) showed that muscles perfused with KPS-1 had significantly less apoptosis when compared with NS as measured by percent positive cells (Figure 5G) (0.49 ± 0.58 versus 2.12 ± 0.88; *P* < 0.001). In addition, the KPS-1 perfused muscle showed no significant differences in TUNEL staining when compared with naive nonperfused muscle (0.38 ± 0.49). CC3 staining (Figure 5 D-F) was also significantly decreased in KPS-1 perfused tissues compared with NS-perfused muscle (Figure 5H) (7.61 ± 1.48 versus 12.8 ± 4.78; *P* = 0.045).

## DISCUSSION

VCA transplantation is currently limited by high rates of rejection and complications secondary to long-term immunosuppression therapy.^31^ Suboptimal outcomes are partially due to the susceptibility of VCAs to cold ischemic injury and IRI. The novel storage approach explored in our study could potentially limit VCA damage during cold storage, greatly improve outcomes, and increase utilization of this treatment, especially for populations such as military personnel injured in the field who may require extended VCA storage time.

Here, we demonstrate that 24-h machine perfusion using KPS-1 did not result in a significant increase in limb weight, muscle edema, or muscle apoptosis. This is further supported by the ability to perform orthotopic hindlimb transplantation on limbs perfused with KPS-1, but not NS, owing to excessive edema. Our findings indicate that the perfused graft may be a viable candidate for transplantation, despite being subjected to a period of ischemic cold storage that surpasses the known critical ischemic point for skeletal muscle.

Previous studies investigating ex vivo perfusion in the context of VCA transplantation have highlighted the issue of increased edema in grafts following perfusion, limiting the time of HMP storage to 6 h. Increased edema is associated with higher instances of graft failure and rejection.^32,33^ Given these findings, our study closely examined limb edema following HMP with either KPS-1 or NS. In contrast to earlier studies, a key outcome seen with our perfusion system is that 24-h perfusion with KPS-1 does not cause a significant increase in edema relative to naive tissue, as measured by weight gain and interfascicular space. Additionally, edema was significantly decreased in limbs perfused with KPS-1 compared with those perfused with NS. It has been hypothesized that the edema observed during ex vivo perfusion is caused by diffusion of the perfusion solution into the interstitial space, suggesting a significant role for an optimized preservation solution.^34^

One of the main goals of this study was to mitigate cell death while extending ex vivo storage past the known critical point of ischemia in the muscle. It is likely that the most significant factor contributing to excessive cell death in ex vivo machine perfusion is the perfusion solution itself.^32,33,35^ Although not a clinical standard, NS has previously been used in practice for the flushing of grafts; however, our results show that it causes extensive damage to skeletal muscle when used for VCA HMP storage. In contrast, KPS-1 demonstrated the ability to significantly reduce TUNEL staining, CC3 staining, and histological markers of apoptosis and necrosis compared with NS. This could be attributed to the composition of electrolytes in KPS-1, colloids such as hydroxyethyl starch and sodium gluconate, and other additives that have been shown to suppress inflammatory responses and reduce IRI.^21,36^

Our study showed insignificant amounts of edema, apoptosis, and necrosis using KPS-1 even after prolonged storage at 24 h. Numerous other studies have shown much shorter storage times and greater amounts of apoptosis, necrosis, and edema using alternative perfusion solutions such as BSA- or DMEM-based solutions.^11,32,33^ Although there is currently no universally accepted perfusion solution for VCA transplantation, our study indicates there is the potential for KPS-1 to fill this gap.

The use of heparinized NS as a perfusion solution during prolonged ischemic conditions proved to be damaging and largely detrimental. In clinical practice, NS is often used in numerous medical contexts including flushing of allografts and other tissues, in post-surgical infusions for volume replacement, resuscitation, and short-term storage of vessel grafts. Recent studies have shown that NS can cause metabolic acidosis, acute kidney injury, gastrointestinal edema, and significant damage to endothelial vascular function.^37-39^ The negative impact of NS described in previous studies corroborates the findings of our study and suggests that the use of NS flushes to prepare VCA and other organs for transplantation should be reconsidered, especially if prolonged cold storage is logistically required.

One of the greatest challenges of this study was to optimize the conditions of the perfusion apparatus to overcome the limitations of preservation solution delivery. This included maintaining a flow rate that enabled complete perfusion of the hindlimb while mitigating the consequences of higher perfusion rates, including edema and swelling.^40^ While other studies have proposed flow rates ranging from 0.1 to 1 mL/min, the model we present in this study utilizes a constant flow rate of 0.2 mL/min. Vascular resistance and pH did not change significantly during KPS-1 perfusion, and although there was increased variation with NS perfusion, the clinical relevance of this is not yet understood. In addition, there was no evidence that the flow rate used in our experiments influenced the amount of weight gained in the hindlimbs.

Different patterns of perfusion, such as pulsatile flow to mimic the physiological rhythm of blood flow throughout the body, have also been suggested as methods to enhance tissue perfusion.^41^ Although relatively new in the context of VCA perfusion, the incorporation of pulsatile flow shows potential to outperform constant flow and is an area of interest for future studies. Furthermore, an optimal flow rate/pattern has not yet been defined, as it can vary depending on the perfusion solution, tissue, and characteristics of the perfusion system itself.^11,32,33^ However, our HMP model shows that it is possible to achieve complete distribution of KPS-1 solution without significantly increasing edema in rat hindlimb VCAs. Therefore, there is potential to further improve this system and its storage outcomes through the addition of cell death inhibitors and other therapeutic agents.

### Limitations

While the number of limbs perfused in each group is small, the amount of data was sufficient to generate the applicable models and to compare results with appropriate statistical tests. Mixed model analysis was used to account for measurements that are not independent and to evaluate potential changes over time. However, we cannot rule out type II error based on this sample size and that nonsignificant findings could be a result of lack of sufficient sample size.

Although VCA transplantation involves multiple tissue types, our study focused only on the effects on skeletal muscle. In VCAs, skeletal muscle is the most dominant and metabolically active tissue. Therefore, it is also the most susceptible to injury during prolonged ischemic storage.^32,36,42^ Other studies have shown that irreversible cell damage can occur in as little as 4 h in skeletal muscle; however, this is not the case in tissues such as nerves, skin, and bone, which reach a critical ischemic time at 8, 13, and 96 h, respectively.^17^ Additionally, we recognize that the highly immunogenic nature of skin can greatly contribute to graft rejection. This is a focal point in many studies, and although it is not included in this report, it serves as a significant area of interest for future studies.

## CONCLUSIONS

Twenty-four-hour ex vivo HMP of rodent hindlimbs using KPS-1 as a perfusion solution is feasible, resulting in a potentially viable graft for transplantation. Machine perfusion with KPS-1 solution significantly reduced tissue weight and edema in HMP-perfused rat hindlimbs when compared with NS-perfused tissue. Furthermore, donor hindlimbs perfused with KPS-1 showed reduced histological markers of apoptosis and necrosis when compared with NS-perfused tissue. Future studies will focus on posttransplant functional and survival applications as well as further optimization of our perfusion solution through the addition of apoptosis and necrosis inhibitors.

## ACKNOWLEDGMENTS

The authors thank Organ Preservation Systems for their generous donation of the KPS-1 solution used in this study. Also, the authors thank Paula Arrowsmith for expert histological processing of the tissues, Niyati Nakra for technical assistance, and Robert Plentor for advice on setting up the perfusion system. Additionally, the authors acknowledge Nathaly Limon De La Rosa, Caitlin Blades, and Li Lu for critical review of the article and Zhiying You for statistical advice.
